# Acromegaly and the thyroid gland

**DOI:** 10.1186/1756-6614-8-S1-A20

**Published:** 2015-06-22

**Authors:** Andrzej Lewiński, Magdalena Marcinkowska

**Affiliations:** 1 Department of Endocrinology and Metabolic Diseases, Medical University of Lodz, Lodz, Poland; 2 Department of Endocrinology and Metabolic Diseases, Polish Mother’s Memorial Hospital – Research Institute, Lodz, Poland

## 

Acromegaly is a chronic disease caused by hypersecretion of growth hormone (GH), most frequently from a pituitary somatotropic adenoma. Its prevalence was estimated at 60–70 cases per million people, but in recent years it seems to be higher (even 86 cases per million). Approximately 3-4 new cases of acromegaly are annually diagnosed per million people.

In acromegalic patients, the mortality rate is 2-4 times higher than in the general population. The most common causes of death in patients in question are cardiovascular and/or respiratory complications, or neoplastic diseases.

Data indicating the increased risk of the development of benign and malignant tumors of various organs, particularly of the colon, thyroid gland, breast, and prostate, are reported in numerous studies. An elevated level of IGF-I seems to be responsible for the increased risk of cancers. It is to be recalled that IGF-I is a mitogenic, anti-apoptotic and angiogenesis-promoting factor.

Prevalence of cancers in acromegalic patients remains controversial: some authors describe the increased prevalence, in contrast, others do not. The difference among studies may be due to a low incidence of acromegaly *per se*, retrospective nature of studies or to differences in study designs. In most studies, patients with cancers diagnosed prior to acromegaly were excluded [1].

The presence of IGF-I receptors was shown in both normal and neoplastic thyroid tissue in humans, a long time ago. There are numerous scientific evidence that IGF-I reveals an important, TSH–independent effect in growth processes in humans thyroid [2,3]. Moreover, there are a lot of studies describing the increased prevalence of goitre (both diffuse and nodular) in acromegalic patients, and many authors have demonstrated a positive correlation between the thyroid volume and serum IGF-I concentration.

Large meta-analysis published recently by Wolinski et al. [4] has confirmed that both thyroid nodular disease and thyroid carcinoma are significantly more frequent in acromegalic patients than in general population (Table 1).

**Table 1:** Studies with control group included in meta-analysis – the table taken from the study by Wolinski et al. [4], modified. Asterisk (*) - persons with non-functioning or Prl secreting adenomas.

Studies with control group	Acromegalic patients	Cases of thyroid carcinoma in group of acromegalic patients	Control group	Cases of thyroid carcinoma in control group	*Odds ratio*
dos Santos et al., *Pituitary 2013;16:109-114*	124	9 (7.25%)	263 (not specified)	2 (0.76%)	9.5 (2.2-48.0)

Herrmann et al., *Clin Endocrinol Diabet. 2004;112:225–230;* retrospective	73	4 (5.5%)	199 (healthy volunteers)	0	25.8 (1.4-486.0)

Gasperi et al., *J Endocrinol Invest.* 2002;25:240-245	258	3 (1.16%)	150*	1 (0.66%)	1.7 (0.2-16.9)

Popovic et al. Clin Endocrinol (Oxf) 1998;49: 441–445; retrospective	220	3 (1.36%)	248*	0	8.0 (0.4-155.7)

Barzilay et al., *Arch Intern Med. 1991;151:1629–1632*; retrospective	87	2 (2.3%)	198*	0	11.6 (0.6-244.4)

Total					7.9 (2.8-22.0)

Accordingly, these results demonstrate that the repeated thyroid ultrasound (US) examination and careful evaluation of possible lesions (together with cytological assessment) should be important part of follow-up in patients with acromegaly. Wolinski et al. [4] documented that in newer studies on acromegalic subjects, thyroid disorders were reported more frequently - in studies published after year 2008, thyroid nodular disease occurred in about 65% of patients whereas in older studies approx. in 54%. Similar phenomenon could be recorded in case of thyroid carcinomas – 6% patients in newer reports published after 2008 vs. 3% in older studies, published before 2008. These results speak for the hypothesis that the improvement in diagnostic methods and therapy of acromegaly extends the survival time of patients, what - in turn - increases the prevalence of benign and malignant neoplasms possible to detect.

Result of selected studies on acromegaly and thyroid disorders, published in recent years, are presented in Tables 2 and 3.

**Table 2:** Results of the retrospective study by Turkish authors [1], including 64 acromegalic patients who were subjected to thyroid US examination and thyroid function tests (distribution of thyroid diagnoses).

Multinodular goitre	Simple nodule	Toxic multinodular goitre	Hürthle cell adenoma	Diffuse goitre	Thyroid carcinoma	No thyroid disease
31 (48.4%)	6 (9.4%)	1 (1.6%)	1 (1.6%)	9 (14.1%)	5 (7.8%)	11 (17.1%)

**Table 3:** Results of study from Brasil, including 106 acromegalic patients, who were subjected to thyroid US examination [5]. In 4 patients (3.8%) thyroid carcinoma was diagnosed in cytological examination (2 cases - multifocal papillary thyroid carcinoma, 1 - papillary microcarcinoma, 1 - papillary variant of follicular thyroid carcinoma).

Multinodular goitre	Simple nodule	Unspecific abnormalities (colloid cyst or heterogenous texture)	Diffuse goitre	Normal US
34 (32.5%)	8 (7.5%)	22 (20.6%)	11 (10.4%)	31 (29%)
